# Self-Powered Flexible Blood Oxygen Monitoring System Based on a Triboelectric Nanogenerator

**DOI:** 10.3390/nano9050778

**Published:** 2019-05-21

**Authors:** Huamin Chen, Yun Xu, Jiushuang Zhang, Weitong Wu, Guofeng Song

**Affiliations:** 1 Institute of Semiconductors, Chinese Academy of Sciences, Beijing 100083, China; chenhuamin@semi.ac.cn (H.C.); jszhang@semi.ac.cn (J.Z.); wuweitong@semi.ac.cn (W.W.); sgf@semi.ac.cn (G.S.); 2 College of Materials Science and Opto-Electronic Technology, University of Chinese Academy of Sciences, Beijing 100049, China; 3 Beijing Key Laboratory of Inorganic Stretchable and Flexible Information Technology, Beijing 100083, China

**Keywords:** triboelectric nanogenerator, blood oxygen, self-power, flexible electronics

## Abstract

Flexible optoelectronics based on inorganic functional components have attracted worldwide attention due to their inherent advantages. However, the power supply problem presents a significant obstacle to the commercialization of wearable optoelectronics. Triboelectric nanogenerator (TENG) technology has the potential to realize self-powered applications compared to the conventional charging technologies. Herein, a flexible self-powered blood oxygen monitoring system based on TENG was first demonstrated. The flexibility of the TENG is mainly due to the inherent properties of polydimethylsiloxane (PDMS) and the continuously undulating surface of crumpled gold (Au) and the rough surface on the electrode and PDMS effectively increased the output performance. The output voltage, output current density, and power density were 75.3 V, 7.4 μA, and 0.2 mW/cm^2^, respectively. By etching the sacrificial layer, we then derived a flexible blood oxygen and pulse detector without any obvious performance degradation. Powered by the TENG, the detector is mounted onto the thumbnail, from where it detects a stable photoplethysmography (PPG) signal which can be used to calculate the oxyhemoglobin saturation and pulse rate. This self-powered system provides a new way to sustainably monitor physiological parameters, which paves the way for development of wearable electronics and battery-free systems.

## 1. Introduction

Flexible and wearable optoelectronics based on inorganic functional components have become the research focus of next-generation electronics due to their inherent advantages of portability, versatility, and comfortability. They have been applied in various areas, including integrated circuits [1,2], display and sensing [3,4,5,6], epidermal electronics [7,8], and energy harvesters [9,10]. Especially in the health monitoring field, many studies and prototype devices have been reported [11,12,13,14]. Despite major breakthroughs in many areas, such as new material development [15,16], mechanics [17,18], and structural design [19,20], the power supply problem remains one of the biggest obstacles to the commercialization of wearable optoelectronics.

The triboelectric nanogenerator (TENG) is a promising energy harvester based on triboelectrification and electrostatic induction [21] and can harvest mechanical energy from daily activities such as body motion, to power low-power-consumption optoelectronics. Compared to conventional charging technologies, TENG can indeed solve the power supply problem on account of its high output performance [22,23], low cost [24], easy fabrication process [25], and intrinsic flexibility [26], which is an indispensable factor. Many self-powered applications have been demonstrated, such as self-powered pacemakers [27], self-powered electrochemistry [28], and self-powered sensors [29,30,31]. Nevertheless, a flexible self-powered system for health monitoring has rarely been reported.

In this work, we firstly demonstrate a flexible self-powered blood oxygen monitoring system based on a TENG. The stretchable and flexible TENG was comprised of a crumpled gold (Au) electrode and polydimethylsiloxane (PDMS) triboelectric layer. The flexibility of PDMS and the unique crumpled structure provides strong flexibility for the TENG. The rough nanostructure on the electrode and PDMS effectively enhances the output power of the TENG. The output voltage, output current density, and power density were 75.3 V, 7.4 μA, and 0.2 mW/cm^2^, respectively. Peeling off the rigid substrate results in a flexible blood oxygen detector without any significant performance degradation. Powered by the TENG, a stable photoplethysmography (PPG) signal was achieved by mounting the detector on the thumbnail to detect and calculate the oxyhemoglobin saturation and pulse rate. The flexible self-powered blood oxygen monitoring system has the potential to sustainably monitor pulse oximetry. This research demonstrates a novel method toward self-powered detection of oxyhemoglobin saturation, which paves the way for the development of flexible and battery-free electronics.

## 2. Materials and Methods

**Figure 1:**
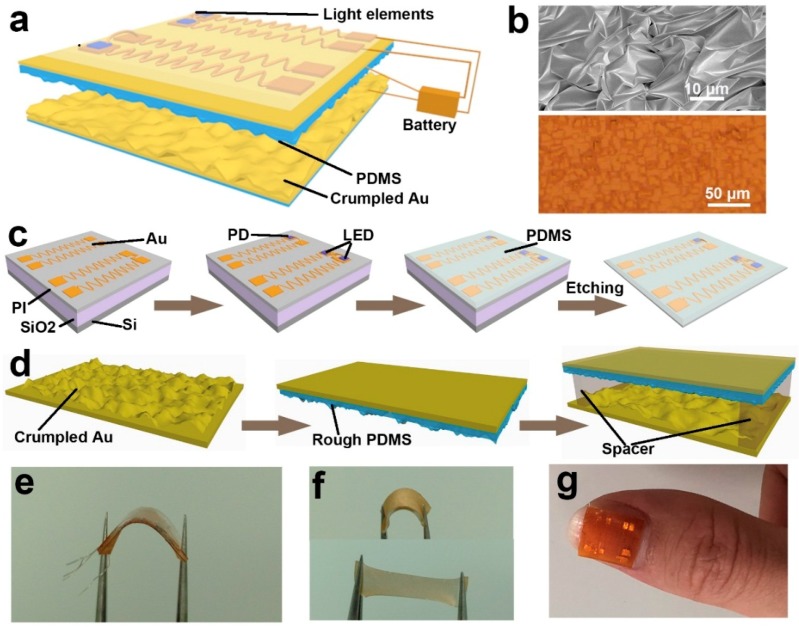
The self-powered flexible blood oxygen monitoring system. (**a**) The schematic illustration of the self-powered flexible blood oxygen monitoring system. (**b**) The high magnification SEM image of the crumpled Au electrode (upper) and 3D image of the PDMS (lower). (**c**) The schematic fabrication process of the flexible blood oxygen device. (**d**) The construction process of the TENG. (**e**) A photograph of the flexible blood oxygen detector. (**f**) The flexibility and stretchability of the TENG. (**g**) A photograph of the flexible blood oxygen system attached on the finger.

Figure 1a shows the schematic structure of the self-powered flexible blood oxygen system. This flexible system mainly consists of a self-powered unit and blood oxygen detector unit. The self-powered unit is made up of a crumpled Au-based TENG and an energy storage unit. The blood oxygen detector unit is a thin film with a thickness of about 0.4 mm. Figure 1b shows the crumpled morphology of the Au electrode, which can effectively enhance the output performance and the stretchability of the TENG. The lower panel of Figure 1b shows the rough surface on PDMS. The schematic fabrication process of the flexible blood oxygen detector is shown in Figure 1c. Firstly, a SiO_2_ sacrificial layer with a thickness of 1 μm was deposited on the Si substrate by inductively coupled plasma chemical vapor deposition (ICPCVP, Plasmalab System 100, Oxford Instruments, UK). Then, the PI (Polyimide, YiDun, China) with a thickness of 1 μm was spin-coated onto the SiO_2_, followed by the deposition of Au (500 nm). The Au pattern was defined by photolithography (MA6, SUSS, Germany). The width of the interconnect wires was 30 μm, and the Au pad was 1 mm × 1 mm. Then, our designed ultrathin light-emitting diodes (LEDs) and photodetector (PD) were transferred onto the Au pad. The liquid PDMS (Sylgard 184, Dow Corning, USA) was spin-coated onto the top and cured to form an enclosure. Finally, the sacrificial layer was etched by hydrofluoric acid (HF), so the thickness of the whole blood oxygen detector was about 0.4 mm. The construction process of the TENG is displayed in Figure 1d. The crumpled Au electrode was formed by the physical method in our previous work [32]. The $\varepsilon_{pre}$ is defined as $\varepsilon_{pre}=L_{pristine}/L_{crumpled}$, where $L_{pristine}$ and $L_{crumpled}$ are the pristine and crumpled length of Au, respectively. In addition, a rough nano/microstructure was introduced onto the surface of PDMS by spin-coating PDMS onto a rough substrate, as shown in Figure 1b. Spacers with a thickness of 2 mm were used to connect the two thin films. The area of the TENG was 1 cm × 1 cm. Figure 1e exhibits the flexible blood oxygen detector with a small bending radius. The flexibility of the device enables it to tightly mount on a fingernail. The flexibility and the stretchability of TENG are shown in Figure 1f. The mechanical properties enable the TENG to conformably contact the skin. More importantly, the TENG can harvest energy from body motion, such as finger bending. Figure 1g shows the photograph of the flexible blood oxygen system attached on a finger.

**Figure 2:**
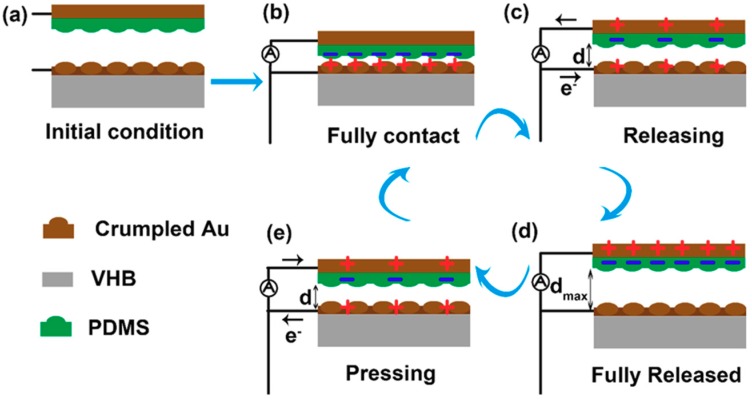
The working mechanism of the TENG. (**a**) The initial condition. (**b**) The device under full contact. (**c**) Releasing the device. (**d**) The device is fully released. (**e**) Pressing the device.

The basic working mechanism of the TENG is explained in Figure 2. In the initial condition (Figure 2a), the upper film and the bottom film are separated. The two films are in full contact under stress and charges are formed on the contact surface of the two films (Figure 2b). As the force is released, the electrons will flow from the upper electrode to the bottom electrode (Figure 2c). The device returns to its original state as the force is fully released (Figure 2d). Then, upon pressing the device, the electrons will flow from the bottom electrode to the upper electrode (Figure 2e). Obviously, the output performance is largely dependent on the surface charge density. So the rough morphology introduced on the surface of the Au and PDMS can largely enhance the output performance.

## 3. Results and Discussion

**Figure 3:**
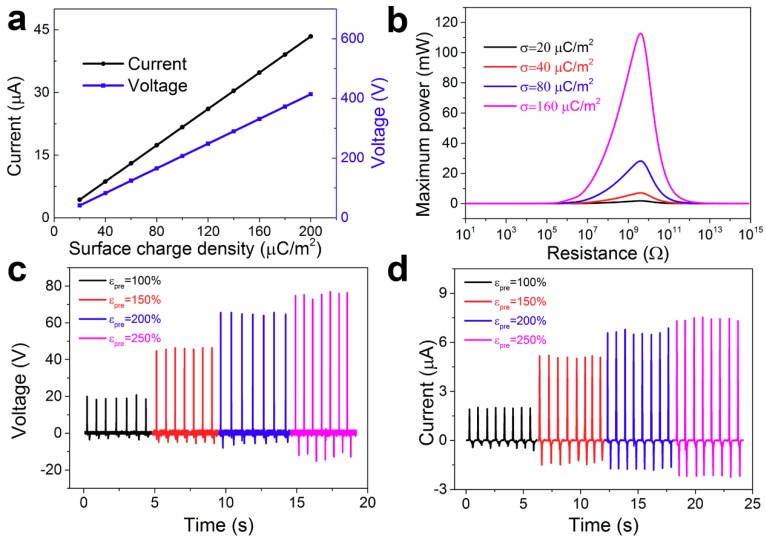
The output performance of the TENG. (**a**) The simulated relationship of output current and output voltage with the surface charge density. (**b**) The simulated relationship of maximum power with the resistance under different surface charge densities. (**c**) The measured output voltage of TENG with various $\varepsilon_{pre}$. (**d**) The measured output current of TENG with various $\varepsilon_{pre}$.

To explicitly understand the effect of the surface charge density on the output performance, we calculated and simulated the relationship between the charge density and the output current and output voltage, as shown in Figure 3a. Clearly, the output current and output voltage were nearly proportional to the surface charge density. The maximum power was mainly dependent on the output current, so the maximum power increased rapidly with increasing the surface charge density, as shown in Figure 3b. It is an effective method to improve the power density. The effect of rough structure on the output performance was studied, as shown in Figure 3c,d. The output voltage was measured under the resistance of 10 MΩ. The output voltage with different $\varepsilon_{pre}$ ($\varepsilon_{pre}\;$= 100%, 150%, 200%, 250%) was 19.2 V, 45.9 V, 64.5 V and 75.3 V, respectively. The output current was 2.0 μA, 5.1 μA, 6.6 μA, and 7.4 μA, respectively. The device presented a four times increase in output voltage current, and thus power was increased by an order of magnitude.

**Figure 4:**
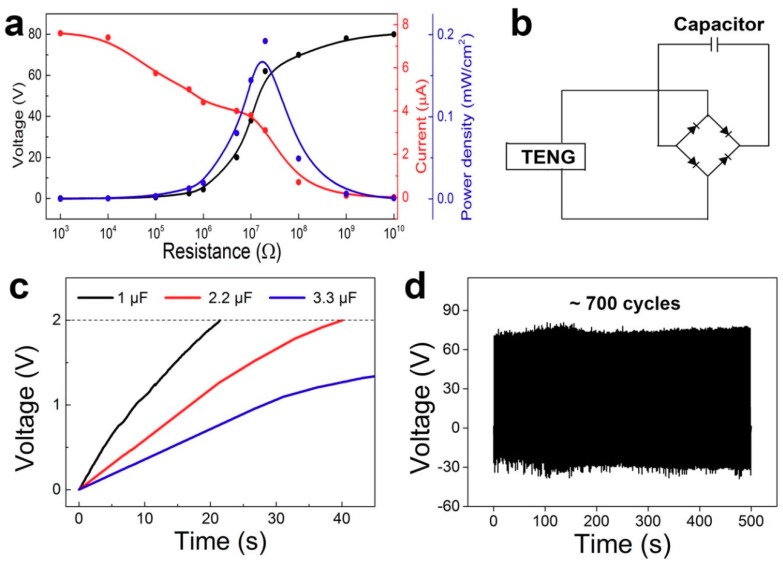
The chargeability of the TENG. (**a**) The relationship between the output performance and the load resistance. (**b**) The schematic diagram of the charging circuit. (**c**) The charging curves of different capacitors (1 μF, 2.2 μF and 3.3 μF). (**d**) The endurance test of the TENG.

The chargeability of the TENG is characterized in Figure 4. The maximum output power is one of the most important parameters for evaluation of the chargeability. The relationship between the power density and the load resistance as measured is shown in Figure 4a. The output voltage increased with the resistance, but the output current showed a contrary tendency. We can see the power density reached a maximum value of 0.2 mW/cm^2^ under a resistance of 20 MΩ. The output current of the TENG is an alternating current, so it should be used to charge the capacitor through a rectifying circuit. The schematic charging circuit is shown in Figure 4b. Three different capacitors (1 μF, 2.2 μF and 3.3 μF) were charged by the TENG. The 1 μF and 2.2 μF capacitors were charged to 2 V in 20 s and 40 s, respectively, as shown in Figure 4c. It is noticed that the charging rate was largely influenced by the rectifying circuit, so we can increase the chargeability by adapting a more effective circuit. The endurance test of the TENG is shown in Figure 4d. The output voltage was measured under a cycled compressive force of about 30 N at a frequency of 1.4 Hz. We can see that the output performance was stable in about 700 cycles. These abilities give the TENG high application value for self-powered units.

**Figure 5:**
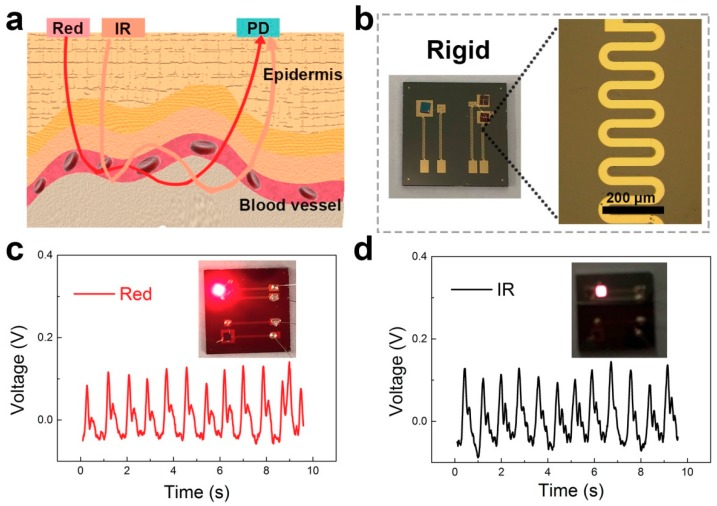
The blood oxygen signal of the rigid device. (**a**) The detection mechanism of the blood oxygen detector. (**b**) The 3D graphs of the device structure. (**c**) The photoplethysmography (PPG) signal of the red LED. (**d**) The PPG signal of the IR LED.

The detection mechanism of the oxygen blood detector is schematically shown in Figure 5a. The light emitted from the LED passes through the epidermis, and received by the PD after scattering, penetration and reflection. The reflected light received by the PD contains information about the hemoglobin. The blood oxygen signal is obtained by PPG. A control experiment of the blood oxygen signal was measured by a rigid oximeter. Images of the patterns are shown in Figure 5b. The wave structure of the interconnect lines can sustain strain in the device which makes it flexible. The width of the line is 50 μm. We obtained the blood oxygen signal by using red and IR light sources, respectively, as shown in Figure 5c,d. In general, the IR signal is stronger than the red signal due to its stronger penetrability. The periodic waveform represents the pulse rate. The blood oxygen value can be deduced from the peak-valley voltage. The signal intensity and stability are greatly affected by the light path. Furthermore, the sustainability and portability of the rigid device remains to be improved.

**Figure 6:**
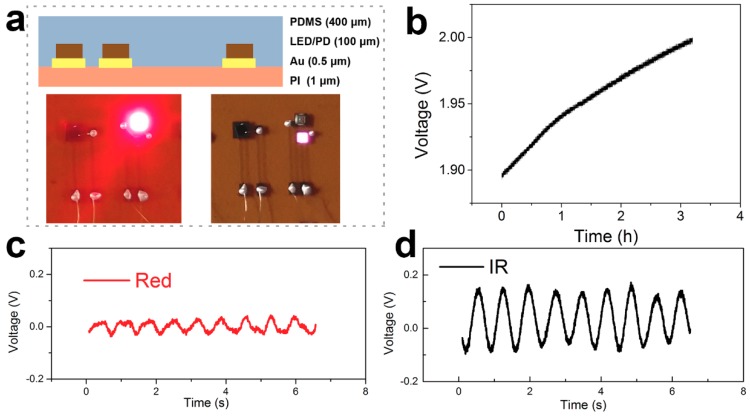
Characterization of the flexible blood oxygen detection system. (**a**) The images of the flexible device structure. (**b**) The battery charged by the TENG. (**c**) The blood oxygen signal of the red LED. (**d**) The blood oxygen signal of the IR LED.

According to the above experimental results, we assembled the TENG and flexible blood oxygen detector into a self-powered flexible blood oxygen system. The results are shown in Figure 6.The device structure of the flexible blood oxygen detector is illustrated in detail in Figure 6a. The total thickness of the flexible device is about 0.4 mm. The lower images show the LEDs lit up by the battery. Figure 6b shows the charging curve of the battery. The battery can be charged up to 2.0 V from 1.9 V in about 3 h. This voltage is sufficient to light up the flexible LEDs. The TENG can harvest mechanical energy from our daily activity to power the flexible device. More importantly, we can shorten the charge time by designing a matched battery and high-efficient rectifying circuit. The blood oxygen signal of the red LED and IR LED are shown in Figure 6c,d. We can see an obvious periodic waveform which can be used to calculate the oxyhemoglobin saturation. However, it should be noted that the measured secondary peak seen in Figure 5c,d subsequently disappeared, as shown in Figure 6c,d. On the one hand, there was unavoidable performance degradation of the flexible detector. On the other hand, we pressed hard on the rigid detector to measure the PPG signal. The strong force resulted in a shorter light path, which provided more abundant information. Fortunately, it did not affect our calculation. This self-powered system attached to a fingertip can sustainably monitor our health index without intense discomfort. This realization of a self-powered detector can pave the way for development of flexible electronics and battery-free electronics.

## 4. Conclusions

In conclusion, we firstly demonstrated a flexible self-powered blood oxygen monitoring system based on a TENG. The flexible TENG was based on a crumpled Au electrode and rough PDMS triboelectric layer which could provide strong flexibility for the whole device. The rough nanostructure on the electrode and PDMS can also effectively enhance the output performance of TENG. The output voltage, output current density, and power density of the TENG ($\varepsilon_{pre}$ = 100%) were 75.3 V, 7.4 μA/cm^2^, and 0.2 mW/cm^2^, respectively. The flexible blood oxygen detector can be obtained by removing the sacrificial layer. Powered by the TENG, the stable PPG signal was achieved by mounting the detector on the thumbnail. The flexible self-powered blood oxygen monitoring system has the potential to sustainably monitor our pulse oximetry without intense discomfort. This research demonstrates a novel method toward self-powered detection of oxyhemoglobin saturation, which paves the way for the development of flexible and battery-free electronics.
